# Pregnancy incidence, outcomes and associated factors in a cohort of women living with HIV/AIDS in Rio de Janeiro, Brazil, 1996-2016

**DOI:** 10.1590/0102-311XEN232522

**Published:** 2023-07-17

**Authors:** Rosa Maria Soares Madeira Domingues, Marcel de Souza Borges Quintana, Lara Esteves Coelho, Ruth Khalili Friedman, Angela Cristina Vasconcelos de Andrade Rabello, Vania Rocha, Beatriz Grinsztejn

**Affiliations:** 1 Instituto Nacional de Infectologia Evandro Chagas, Fundação Oswaldo Cruz, Rio de Janeiro, Brasil.

**Keywords:** Pregnancy, HIV Infections, Cohort Studies, Gravidez, Infecções por HIV, Estudos de Coortes, Embarazo, Infecciones por HIV, Estudios de Cohortes

## Abstract

The aim of this research was to analyze pregnancy incidence and associated factors in a cohort of 753 women living with HIV/AIDS (WLWHA) in Rio de Janeiro, Brazil, from 1996 to 2016. Women aged 18-49 years who were not on menopause (surgical or natural) and did not have a tubal ligation were eligible for the study. Data were collected by medical professionals during initial and follow-up visits. Person-time pregnancy incidence rates were calculated throughout the follow-up period. Pregnancy incidence-associated factors were investigated by univariate and multiple analyzes, using an extension of the Cox survival model. Follow-up visits recorded 194 pregnancies, with an incidence rate of 4.01/100 person-years (95% CI: 3.47; 4.60). A higher pregnancy incidence was associated with CD4 nadir ≥ 350 cells/mm³, use of an antiretroviral regimen not containing Efavirenz, and prior teenage pregnancy. In turn, women with a viral load ≥ 50 copies/mL, age ≥ 35 years old, with two or more children and using a highly effective contraceptive method showed a lower incidence. Results showed a significant reduction in pregnancy incidence after 2006, a significant reduction in female sterilization from 1996 to 2016, and a high rate of cesarean sections. The association found between pregnancy incidence and the use of contraceptive methods and virological control markers suggests a good integration between HIV/AIDS and reproductive health services. The high rate of cesarean section delivery indicates the need to improve childbirth care.

## Resumo

O objetivo deste estudo foi analisar a incidência de gravidez e fatores associados em uma coorte de mulheres vivendo com HIV/aids (MVHA) no Rio de Janeiro, Brasil. Foi realizada uma coorte clínica com 753 MVHA entre 1996 e 2016. Mulheres com idade entre 18-49 anos que não estavam na menopausa natural ou cirúrgica e que não tinham laqueadura tubária foram elegíveis para o estudo. Os dados foram coletados durante as consultas iniciais e de acompanhamento por profissionais médicos. As taxas de incidência por pessoa-tempo foram calculadas durante todo o período de acompanhamento. Análises univariadas e múltiplas foram realizadas para investigar fatores associados à incidência de gravidez, utilizando uma extensão do modelo de sobrevida de Cox. Foram registradas 194 gestações durante as consultas de acompanhamento, com uma taxa de incidência de 4,01/100 pessoas-ano (IC95%: 3,47-4,60). Uma maior incidência de gravidez foi associada a nadir CD4 ≥ 350 células/mm³, terapia antirretroviral sem Efavirenz e gravidez anterior na adolescência, enquanto uma menor incidência foi observada em mulheres com carga viral ≥ 50 cópias/mL, idade ≥ 35 anos, com dois ou mais filhos e que usavam um método contraceptivo altamente eficaz. Houve uma redução significativa na incidência de gestações após 2006, uma redução significativa na esterilização feminina entre 1996 e 2016 e uma alta taxa de cesarianas. A associação entre a incidência de gestações e o uso de métodos contraceptivos e marcadores de controle virológico sugere uma boa integração entre HIV/aids e serviços de saúde reprodutiva. A alta taxa de cesarianas indica a necessidade de melhoria na assistência ao parto.

## Resumen

El objetivo de este estudio fue analizar la incidencia del embarazo y los factores asociados en una cohorte de mujeres que viven con VIH/SIDA (MVHA) en Río de Janeiro, Brasil. Se realizó una cohorte clínica con 753 MVHA entre 1996 y 2016. Las mujeres de 18-49 años que no estaban en la menopausia natural o quirúrgica y que no tenían ligadura de trompas fueron elegibles para el estudio. Los datos se recopilaron durante las consultas iniciales y de seguimiento por parte de profesionales médicos. Las tasas de incidencia por persona-tiempo se calcularon durante todo el período de seguimiento. Se realizaron análisis univariados y múltiples para investigar los factores asociados con la incidencia del embarazo utilizando una extensión del modelo de supervivencia de Cox. Se registraron 194 embarazos durante las consultas de seguimiento, con una tasa de incidencia de 4,01/100 personas-año (IC95%: 3,47-4,60). Una mayor incidencia de embarazo se asoció con nadir CD4 ≥ 350 células/mm^3^, terapia antirretroviral sin Efavirenz y embarazo previo en la adolescencia, mientras que una menor incidencia se observó en mujeres con carga viral ≥ 50 copias/mL, edad ≥ 35 años, con dos o más hijos y que usaban un método anticonceptivo muy eficaz. Hubo una reducción significativa en la incidencia de embarazos después de 2006, una reducción significativa en la esterilización femenina entre 1996 y 2016 y una alta tasa de cesáreas. La asociación entre la incidencia de embarazos y el uso de métodos anticonceptivos y marcadores de control virológico sugiere una buena integración entre el VIH/SIDA y los servicios de salud reproductiva. La alta tasa de cesáreas indica la necesidad de mejorar la asistencia al parto.

## Introduction

Highly active antiretroviral therapy (HAART) has affected the reproductive pattern of women living with HIV/AIDS (WLWHA) by increasing women’s survival and quality of life and reducing mother-to-child HIV infection transmission. While research conducted in the 1990s showed a lower pregnancy incidence and a higher rate of pregnancy terminations in WLWHA ^1^ ^,^ ^2^ ^,^ ^3^ ^,^ ^4^, more recent studies report an increased number of births in this population ^5^, with rates similar to those observed in the general population ^6^ ^,^ ^7^. But the proportions of unplanned pregnancies and induced abortions remain high, stressing the importance of greater integration between clinical care and reproductive health services ^8^.

Brazil is a continental country with an estimated population of 200 million inhabitants and a concentrated HIV epidemic. Although HIV/AIDS detection rate in women showed a 50% reduction in the period 2010-2020 ^9^, the HIV detection rate in pregnant women increased by 30.5%, going from 2.1 cases/1,000 live births in 2010 to 2.7 cases/1,000 live births in 2020. This finding may be explained by the greater diagnosis of HIV infection during prenatal care and improved surveillance of mother-to-child transmission ^9^. Clinical guidelines for preventing mother-to-child transmission have been available in Brazil since the 1990s, and despite reports of implementation flaws of these protocols ^10^ ^,^ ^11^ ^,^ ^12^, the mother-to-child transmission rate decreased in the country, being estimated at 2% in 2011-2012 ^13^, with a 69.7% reduction in the AIDS detection rate in children under 5 in the period 2010-2020 ^9^.

Research on pregnancy incidence in WLWHA in Brazil are scarce. A previous study conducted with data from a cohort of women followed at the Evandro Chagas Brazilian National Institute of Infectious Diseases, Oswaldo Cruz Foundation (INI/Fiocruz), from 1996 to 2003, found a pregnancy rate of 6.9/100 WLWHA, 2.1/100 of induced abortions, and 33.3% of repeat pregnancies ^14^. The authors observed higher pregnancy rates in younger women and in women living with a partner, while highly educated WLWHA using antiretroviral (ART) showed lower rates. Thus, this study sought to analyze pregnancy incidence, outcomes and associated factors during a 20-year follow-up period in the INI/Fiocruz women cohort.

## Methods

### Study design

Clinical WLWHA cohort assisted at INI/Fiocruz, located in Rio de Janeiro, Brazil. The cohort began in 1996 and was paused in December 2016. Inclusion criteria for participation in the cohort consisted of being female at birth, being over 18 years old and having a confirmed HIV infection diagnosis ^15^.

### Inclusion criteria

All women with at least two visits (initial consultation and one follow-up visit) and who, at the initial visit, were under 50 years old and were not on natural menopause (period of no menstrual cycles for more than one year), surgical menopause (total or subtotal hysterectomy and/or bilateral oophorectomy), or had not undergone tubal ligation surgery were eligible.

### Data collection

A team comprising clinicians and gynecologists regularly (annual or biannual consultations) monitored all women, using standardized instruments to collect gynecological and behavioral data. All medical professionals underwent training to standardize data collection ^15^.

Self-reported race/ethnicity, schooling years, lifetime drug use, age at onset of sexual activity, lifetime number of partners and obstetric history, including teenage pregnancy (under 20 years old), were collected at the initial consultation and analyzed as baseline predictors. Information on age, number of children, domestic violence, sexual violence, smoking, alcohol use, viral load, CD4 nadir, ART use and diagnosis of opportunistic disease ^16^ were obtained at the first visit and updated at follow-up. Data regarding marital status and contraceptive use (“no use”; “low efficacy” - natural, male condom, female condom, diaphragm, withdrawal; “high efficacy” - oral hormones, injectable hormonal, IUD; “combined” - high efficacy associated with a barrier method) were collected exclusively at follow-up visits. All data updated during follow-up were analyzed as time-dependent predictors.

As INI/Fiocruz does not provide obstetric care, we collected outcomes of new pregnancies and dates during cohort visits and/or HIV and gynecological routine care after the event. Outcomes were classified into vaginal delivery, cesarean section, spontaneous abortion (including tubal pregnancies) and voluntary termination of pregnancy (induced abortion). Data on natural or surgical menopause and tubal ligation was also verified during follow-up.

Observation of these women began from their inclusion in the cohort, or from the date of termination of pregnancy for those who were pregnant at the initial consultation. During follow-up, new observation periods began after a new pregnancy ended. Follow-up lasted until their final gynecological appointment on December 31, 2016, or when they had natural or surgical menopause, tubal ligation surgery, or turned 50.

### Data analysis

First, we described the women’s social, demographic, clinical and laboratory characteristics at the beginning of follow-up using frequencies and summary measures.

We then described the outcomes of all pregnancies, including those existing at the initial visit, divided into four periods: 1996-2000, 2001-2006, 2007-2010 and 2011-2016. Chi-square test of tendency, with a 0.05 significance level, was used to verify whether there was a change in the type of outcome during these follow-up periods.

Pregnancy incidence during follow-up was estimated by calculating person-time incidence rates (per 100 person-years) throughout the follow-up period and over the four periods defined for the cohort. Changes in the pregnancy incidence rate over the follow-up periods when compared to the 1996-2001 period was estimated by Wald test, with a 0.05 significance level.

Finally, factors associated with the outcome “pregnancy incidence” were investigated using an extension of the Cox survival model ^17^, which allows for the inclusion of time-dependent variables, identifying each patient as a cluster (inclusion of more than one pregnancy per patient) and stratifying the baseline incidence curve by the total number of pregnancies over time. Time-dependent variables used the record closest to the outcome (pregnancy), considering a maximum window of one year. The univariate and multiple analyzes performed used the free software R version 4.0.5 (http://www.r-project.org).

The cohort study of WLWHA at INI/Fiocruz was approved by the institution’s Ethics Research Committee. All data confidentiality procedures were adopted and all participants signed an informed consent form upon joining the cohort.

## Results

**Figure 1:**
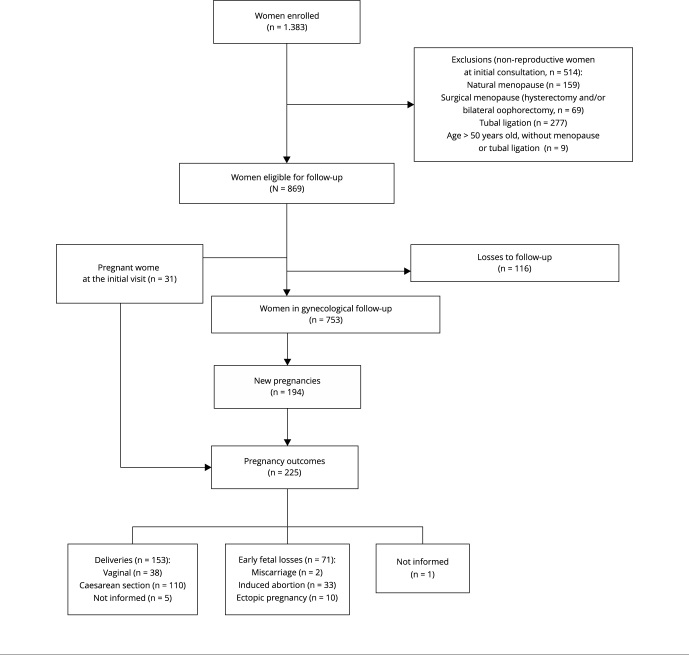
Flowchart of the women cohort for pregnancy outcomes analysis. Rio de Janeiro, Brazil, 1996-2016.

From 1996 to December 2016, a total of 1,383 WLWHA joined the INI/Fiocruz gynecological cohort. Of these, we selected 753 for the present analysis. Figure 1 describes the reasons for exclusion.

Most of the study sample joined the cohort after 2006. Mean age was 33.4 years (standard deviation - SD = 7.8), most reported being mixed race and had more than 8 years of schooling. Nearly 20% were smokers, 38.3% reported consumption of alcoholic beverages and 16.8% the use of some other drug. Almost a third of the women reported past experiences of domestic violence and 13.4% suffered sexual violence. Of the study sample, 18.7% (median age = 16.5 years, interquartile range - IQR: 15; 18) reported onset of sexual activity under the age of 15 and most had more than 5 sexual partners in their lifetime. More than 80% of the women reported a previous pregnancy, 47.1% of which had their first pregnancy in adolescence. Mean number of children was 1.4 (SD = 1.3) and almost half of the sample had a previous abortion. Almost half of the women lived with a partner and used low efficacy contraceptive methods. Most were exposed to HIV through sexual contact, and one woman was exposed by vertical transmission. At initial consultation, 61.4% reported a HIV infection diagnosis of less than 2 years, 24% had already been diagnosed with an opportunistic disease, 62.9% had a viral load ≥ 50 copies/mL, 45.8% CD4 nadir < 200 cells/m³, and 41.8% did not use antiretroviral therapy (Table 1).

**Table 1:** Social, demographic, obstetric, behavioral and virological characteristics of women at entry into the cohort. Rio de Janeiro, Brazil, 1996-2016.

Women characteristics	Total (N = 753)
Enrollment period	
1996-2000	149 (19.8)
2001-2005	138 (18.3)
2006-2010	295 (39.2)
2011-2016	171 (22.7)
Age (years)	
Mean (SD)	33.4 (7.8)
< 20	22 (2.9)
20-34	422 (56.0)
≥ 35	309 (41.0)
Race/Ethnicity	
White	280 (37.7)
Black	179 (24.1)
Mixed	280 (37.7)
Schooling years	
≤ 8	349 (47)
> 8	388 (52.3)
Missing	5 (0.7)
Smoking	155 (20.7)
Alcohol use	287 (38.3)
Drug use	125 (16.8)
Domestic violence	205 (27.7)
Sexual violence	99 (13.4)
Age at first sexual intercourse (years)	
Median (IQR)	16.5 (15; 18)
< 15	141 (18.7)
15-19	487 (64.7)
≥ 20	116 (15.4)
Lifetime partners	
< 5	301 (40)
5-9	229 (30.4)
≥ 10	180 (23.9)
Missing	43 (5.7)
Gravidity	648 (86.1)
Mean (SD)	2.4 (1.9)
Pregnancy in adolescence (< 20 years)	355 (47.1)
Lifetime history of abortion	342 (45.4)
Mean (SD)	0.8 (1.1)
Lifetime history of induced abortion	219 (29.1)
Number of children [mean SD)]	1.4 (1.3)
Live with partner	356 (47.5)
Contraceptive method	
Low efficacy *	368 (49.1)
Hormonal or IUD	58 (7.7)
Dual (hormonal or IUD + barrier)	78 (10.4)
Missing	246 (32.8)
Route of HIV exposure	
Sexual	650 (86.3)
Injection drug use	4 (0.5)
Blood transfusion	12 (1.59)
Vertical transmission	1 (0.1)
Unknown	86 (11.4)
Time since HIV infection diagnosis (years)	
< 2	462 (61.4)
2-4	145 (19.3)
≥ 5	142 (18.9)
Diagnosis of opportunistic disease	181 (24.0)
HIV-1 RNA (copies/mL)	
< 50	136 (18.1)
≥ 50	474 (62.9)
Missing	143 (19)
CD4+ T lymphocyte count nadir (cells/mm^3^)	
Mean (SD)	24.6 (20.7)
< 200	345 (45.8)
200-349	247 (32.8)
350-499	81 (10.8)
≥ 500	78 (10.4)
ART use	
No use	315 (41.8)
HAART with Efavirenz	163 (21.6)
HAART without Efavirenz	206 (27.4)
No HAART	67 (8.9)

ART: antiretroviral; HAART: highly active antiretroviral therapy; IQR: interquartile range; SD: standard deviation.

* Natural, male condom, female condom, diaphragm, withdrawal.

We recorded 225 pregnancies in total, 31 at the initial visit and 194 at follow-up. We observed a significant reduced proportion of women who joined the cohort while pregnant: 11.2% in the first period to less than 3% in subsequent periods (p < 0.01). Of the 178 women who had a pregnancy, 20% were repeat pregnancies (5% with 3 or more pregnancies). Delivery (68%) was the most frequent pregnancy outcome, with 16.9% vaginal deliveries and 48.9% cesarean sections. We recorded 71 early fetal losses (12.4% spontaneous abortion, 14.7% induced abortion, 4.4% ectopic pregnancy). We found no significant differences in pregnancy outcomes during the analyzed periods (Table 2). Ectopic pregnancies increased in 2006-2010, reaching 8.5% of the total outcomes. Half of the ectopic pregnancies were repeat pregnancies, occurring in only two women. Delivery by cesarean section increased in 2000-2006, with subsequent stabilization in the periods 2006-2010 and 2011-2016. Performance of tubal ligation surgery at the time of delivery showed a significant reduction during the analysis period (Table 2).

**Table 2:** Pregnancy outcomes according to follow-up period in the cohort. Rio de Janeiro, Brazil, 1996-2016.

Pregnancy outcome/Follow-up period	1996-2000 (n = 187)	2001-2005 (n = 142)	2006-2010 (n = 304)	2011-2016 (n = 236)	Total (N = 869)	p-value *
	n (%)	n (%)	n (%)	n (%)	n (%)	
Number of pregnancies	51	46	59	69	225	
Deliveries	39 (76.5)	24 (52.2)	40 (67.8)	50 (72.5)	153 (68.0)	0.829
Vaginal delivery	13 (33.3)	2 (8.3)	12 (30.0)	11 (22.0)	38 (24.8)	0.567
Cesarean section	25 (64.1)	20 (83.3)	28 (70.0)	37 (74.0)	110 (71.9)	0.510
Not informed	1 (2.6)	2 (8.3)	0 (0.0)	2 (4.0)	5 (3.3)	
Spontaneous abortion	2 (3.9)	12 (26.1)	8 (13.6)	6 (8.7)	28 (12.4)	0.976
Ectopic pregnancy	1 (2.0)	0 (0.0)	5 (8.5)	4 (5.8)	10 (4.4)	
Induced abortion	9 (17.6)	10 (21.7)	6 (10.2)	8 (11.6)	33 (14.7)	0.163
Outcome not informed	0 (0.0)	0 (0.0)	0 (0.0)	1 (1.5)	1 (0.4)	
Tubal ligation during delivery	21 (53.8)	9 (37.5)	4 (10.0)	10 (20.0)	44 (28.8)	< 0.001

* Chi-square test of tendency, significance level of 0.05. Test not performed when the variables had zero cases in any of the analyzed periods.

The 194 pregnancies observed during follow-up were recorded among 4,841,149 person-years, resulting in a pregnancy incidence rate of 4.01/100 person-years (95% confidence interval - 95%CI: 3.47; 4.60). We found a non-significant difference in the pregnancy incidence rate between the periods 1996-2000 (6.59) and 2001-2005 (3.11, p = 0.342), but a significant reduction to 2.17 in 2006-2010 (p = 0.018) and to 1.49 in 2011-2016 (p = 0.027). The main factors associated with a higher pregnancy incidence rate were CD4 nadir ≥ 350, use of an ARV regimen without Efavirenz, and adolescent pregnancy. Women ≥ 35 years old, with two or more children, who had a viral load ≥ 50 copies/mL, and used a highly effective contraceptive method showed a lower incidence rate (Table 3).

**Table 3:** Univariate and multiple analysis of factors associated with pregnancy incidence. Rio de Janeiro, Brazil, 1996-2016.

Women characteristics	Univariate analysis		Multiple analysis *	
	HR (95%CI)	p-value	HR (95%CI)	p-value
Follow-up period				
1996-2000	1.00			
2001-2005	0.79 (0.49; 1.28)	0.34	1.17 (0.68; 2.00)	0.57
2006-2010	0.58 (0.37; 0.91)	0.02	0.74 (0.40; 1.37)	0.34
2011-2016	0.59 (0.37; 0.94)	0.03	0.55 (0.29; 1.07)	0.08
Age (years)				
< 20	1.00			
20-34	0.45 (0.2; 1.0)	0.05	0.63 (0.27; 1.50)	0.30
≥ 35	0.12 (0.05; 0.28)	< 0.01	0.22 (0.08; 0.56)	< 0.01
Race/Ethnicity				
White	1.00			
Black	0.94 (0.62; 1.44)	0.79		
Mixed	0.72 (0.50; 1.03)	0.07		
Missing	0.95 (0.22; 4.16)	0.95		
Schooling years				
≤ 8	1.00			
> 8	0.92 (0.66; 1.27)	0.60		
Missing	0.98 (0.13; 7.18)	0.98		
Smoking				
No	1.00			
Yes	1.05 (0.72; 1.53)	0.80		
Alcohol use				
No	1.00			
Yes	0.96 (0.70; 1.31)	0.79		
Missing	0.96 (0.21; 4.45)	0.96		
Drug use				
No	1.00			
Yes	1.35 (0.86; 2.13)	0.19		
Missing	0.74 (0.20; 2.72)	0.65		
Domestic violence				
No	1.00			
Yes	0.83 (0.58; 1.19)	0.32		
Missing	0.33 (0.04; 2.54)	0.28		
Sexual violence				
No	1.00			
Yes	1.13 (0.73; 1.75)	0.57		
Missing	1.21 (0.27; 5.42)	0.81		
Pregnancy in adolescence				
No	1.00		1.00	
Yes	1.73 (1.17; 2.56)	0.01	1.65 (1.12; 2.44)	0.01
Missing	1.1 (0.53; 2.30)	0.80	0.57 (0.25; 1.28)	0.17
Age at first sexual intercourse (years)				
< 15	1.00			
15-19	0.65 (0.45; 0.92)	0.02		
≥ 20	0.14 (0.06; 0.30)	< 0.01		
Missing	0.4 (0.14; 1.20)	0.10		
Marital status				
Does not live with a partner	1.00			
Live with a partner	1.55 (1.12; 2.14)	0.01		
Number of children				
Median (IQR)	0.83 (0.68; 1.03)	0.09		
0	1.00			
1	0.64 (0.41; 1.02)	0.06	0.55 (0.36; 0.84)	0.01
≥ 2	0.48 (0.27; 0.83)	0.01	0.43 (0.26; 0.71)	< 0.01
Contraceptive method				
Low efficacy **	1.00		1.00	
Hormonal or IUD	0.24 (0.08; 0.72)	0.01	0.27 (0.10; 0.78)	0.02
Dual (hormonal or IDU + barrier)	0.53 (0.37; 0.76)	< 0.01	0.63 (0.41; 0.99)	0.04
Missing	0.54 (0.33; 0.86)	0.01	0.62 (0.38; 1.02)	0.06
Time since HIV infection diagnosis (years)				
< 2	1.00			
2-4	1.09 (0.71; 1.66)	0.70		
≥ 5	0.94 (0.57; 1.56)	0.81		
Missing	3.02 (1.61; 5.66)	< 0.01		
Diagnosis of opportunistic disease				
No	1.00			
Yes	0.85 (0.58; 1.24)	0.40		
HIV-1 RNA (copies/mL)				
< 50	1.00		1.00	
≥ 50	0.91 (0.67; 1.24)	0.55	0.57 (0.39; 0.85)	0.01
Missing	2.01 (0.87; 4.67)	0.10	0.91 (0.33; 2.55)	0.86
CD4+ T lymphocyte count nadir (cells/mm³)				
Median (IQR)	1.01 (1.01; 1.02)	< 0.01		
< 200	1.00		1.00	
200-349	1.3 (0.90; 1.88)	0.17	1.19 (0.82; 1.73)	0.36
350-499	1.84 (1.17; 2.90)	0.01	2.58 (1.58; 4.23)	< 0.01
≥ 500	2 (1.18; 3.38)	0.01	2.78 (1.57; 4.90)	< 0.01
ARV use				
No use	1.00		1.00	
HAART with Efavirenz	0.13 (0.05; 0.34)	< 0.01	0.19 (0.07; 0.51)	< 0.01
HAART without Efavirenz	2.18 (1.36; 3.51)	< 0.01	3.39 (2.01; 5.73)	< 0.01
No HAART	2.1 (1.12; 3.91)	0.02	1.69 (0.78; 3.67)	0.19

95%CI: 95% confidence interval; ART: antiretroviral; HAART: highly active antiretroviral therapy; HR: harzard ratio.

* Variables that showed statistical significance in the univariate analysis (p < 0.10) and those clinically relevant were included in the multiple analyses. After backwards selection, variables that were statistically significant at the 5% level were included in the final model;

** Natural, male condom, female condom, diaphragm, withdrawal.

## Discussion

Results of this study, which evaluated a WLWHA cohort followed at the largest service for HIV/AIDS care and prevention in Rio de Janeiro, over 20 years (1996-2016), show a pregnancy incidence of 4.01/100 person-years, with a significant reduction after 2006.

Studies conducted from 1996 to 2016 ^4^ ^,^ ^6^ ^,^ ^18^ ^,^ ^19^ ^,^ ^20^ ^,^ ^21^ reported pregnancy incidence ranging from 2.9 per 100 person-years ^19^ to 10.1/100 person-years ^21^. Contrary to our findings, Haddad et al. ^6^, in a study conducted in 9 U.S. centers, reported a non-significant increase in pregnancy incidence in WLWHA and a significant increase in live births (from 2.85 to 7.27/100 person-years) in the periods 1994-1997 to 2006-2012, with similar values in WLWHA and in women not living with HIV/AIDS (WNLWHA) in more recent periods. Studies in Canada ^7^ and Europe ^22^ also report an increased number of pregnancies in WLWHA after the years 2000-2002. One study in Burkina-Faso ^20^ showed an increase in pregnancy incidence in women using ART for longer periods. Moreover, United Kingdom and Ireland ^23^ reported an increase in repeat pregnancies and an increase in repeat pregnancies in women on ART was observed in Latin America and the Caribbean ^24^.

Although we did not assess pregnancy incidence in WNLWHA, to verify whether this outcome would differ between these groups of women, the fertility rates of Brazilian women faced a significant decrease, especially after the 2000s, reaching an average of 1.7 children per woman in 2010-2015 ^25^. The significant reduction in pregnancy incidence observed after 2006 may simply mirror the fertility pattern of the country, with a marked reduction in the South and Southeast regions, where our service is located.

The rate of repeated pregnancies observed was intermediate between that reported by French et al. ^23^, in a U.K. study conducted in 1990-2009 (25.9%), and that reported by Floridia et al. ^26^, in Italy, in the period 2011-2016 (16%). In the latter, women with repeated pregnancies presented more negative outcomes in the first pregnancy than those who did not have a repeat pregnancy. Similarly, we observed that 50% of ectopic pregnancies occurred in only two women, in repeat pregnancies.

Although data on reproductive planning and intention to become pregnant were not available for analysis, the lower pregnancy incidence observed in women who used more effective contraceptive methods and in women with unfavorable viral infection control parameters, such as viral load ≥ 50 copies/mL and nadir of CD4 < 200 cells/mm³, suggest planned pregnancies, considering the reproductive intention and clinical condition of each woman. The higher rate of pregnancies in women taking ART without Efavirenz supports this hypothesis, suggesting the lower prescription of ART regimens with this drug for women who expressed the intention of becoming pregnant, since efavirenz can cause neurological defects in the newborn.

We observed a higher proportion of pregnant women at the initial consultation in the first follow-up period (1996-2000). Information about the time of diagnosis of the infection, whether it occurred during pregnancy or before, is unavailable, and our hypotesis for the higher proportion of pregnant women in the period 1996-2000 is HIV infection diagnosis during prenatal care. Although Brazil had restricted access to serological HIV testing in the late 1990s, the Brazilian Ministry of Health’s guidelines recommended offering HIV testing to all pregnant women during prenatal care since the 1990s. Thus, in a context of limited HIV testing, prenatal care became an opportunity for performing HIV infection diagnosis.

Limited access to testing also explains the late HIV infection diagnosis during the first periods of our cohort: a quarter of the women already had a diagnosis of opportunistic disease when joining and almost 50% had a CD4 nadir below 200 cells/mm³. An analysis including the total number of WLWHA assisted at the INI/Fiocruz cohort identified improved parameters related to HIV infection - such as higher CD4 counts, lower viral load and use of antiretroviral therapy - in more recent years of entry, which the authors attribute to successful national HIV/AIDS control policies ^27^.

Similar to other studies, the factors associated with pregnancy incidence were related both to sociodemographic characteristics, such as age ^1^ ^,^ ^2^ ^,^ ^4^ ^,^ ^6^ ^,^ ^22^ ^,^ ^28^ ^,^ ^29^ ^,^ ^30^, number of children ^1^, and use of contraceptive methods ^6^, and to HIV infection characteristic, such as CD4 nadir ^2^ ^,^ ^6^ ^,^ ^22^ ^,^ ^29^, viral load ^4^, and use of ART medication ^4^ ^,^ ^7^ ^,^ ^31^. These findings are in line with previous studies with WLWHA, which reveal that reproductive decisions are complex and not solely determined by HIV infection aspects ^1^ ^,^ ^6^ ^,^ ^32^.

The higher incidence of pregnancies in women with a history of adolescent pregnancy is consistent with the fertility profile observed in Brazil. Although the total fertility rate is decreasing, fertility in youth under 20 years of age remains high, especially among groups with lower income and schooling, reaching a maximum value in the 20-24 age group. Rather than expressing a desire to have children at this age, this high fertility results from the lack of access to reproductive health services and contraceptive methods ^25^. Early pregnancy can limit achieving a higher educational level and entering and remaining in the labor market, turning motherhood into a strategy of social recognition or even a possible life project in a society with limited possibilities ^33^ ^,^ ^34^. Adolescent pregnancy also affects other Latin American countries, where 20% of births is by adolescent mothers. Reducing adolescent fertility is one of the Pan-American Health Organization’s (PAHO) goals for sustainable development in the period 2018-2030 ^35^.

The ectopic pregnancy prevalence observed was higher than that reported by Stringer et al. ^36^ in Africa (1%), but similar to that observed by research in China (3.4%) ^37^ and the U.S.A. (5%) ^4^. We found a proportion of induced abortions lower than that reported by other studies ^2^ ^,^ ^3^ ^,^ ^4^ ^,^ ^6^ ^,^ ^7^ ^,^ ^22^ ^,^ ^29^ and without a significant reduction in the 20-year period analyzed. Studies conducted in the U.S. and in European countries report a significant reduction in the frequency of induced abortions with the advancement of ART ^2^ ^,^ ^3^ ^,^ ^4^ ^,^ ^5^. However, a U.S. study with a WLWHA cohort found no evidence of a reduction in induced abortions or its association with aspects related to infection control, indicating the need for greater integration with contraception services ^6^.

The increased proportion of cesarean sections from 2001 to 2005 is consistent with existing recommendations at the time of performing cesarean section to reduce the mother-to-child transmission rate ^38^ ^,^ ^39^. Later studies, however, started recommending elective cesarean section only for women with high viral load after the 34th gestational week ^40^ ^,^ ^41^. Such recommendation was not followed by a reduction in the cesarean section rate in more recent periods of our cohort, which remained stable and above 70%. In the U.K., the highest cesarean section rates in WLWHA were observed in 1999, with a significant increase in vaginal deliveries in the period 1999-2006 ^5^. Similarly, France saw an increase from 25% in 2000 to 53% in 2010 in the rate of vaginal births in WLWHA ^40^. Brazil is one of the countries with the highest cesarean rate worldwide, largely determined by non-clinical factors ^42^. The high cesarean section rate observed in this WLWHA cohort may reflect the existing care model, with great flexibility in indicating cesarean sections or even little familiarity with the most recent guidelines on the best mode of delivery for WLWHA. Difficulty in accessing viral load tests may be another explanation. A national, hospital-based study conducted in 2011-2012 showed that 45% of pregnant WLWHA had an undiagnosed viral load at delivery ^13^.

We observed a significant reduction in the proportion of tubal ligation surgery performed during follow-up in the cohort, from 53.8% to 20%, a trend also observed countrywide. Brazil saw a reduction in female sterilization among married women (aged 15-44 years) from 38.5% to 25.9% in the period 1996-2006 ^43^. Sterilization had become the second most frequent contraceptive method in the country, although mostly used by women with lower education and from lower economic classes. In the 2013 *Brazilian National Health Survey*, 25.9% of sexually active women aged 18-49 years reported using surgical methods (male and female), especially women with less education and no health insurance ^44^.

Despite the decline in female sterilization in this group, WLWHA still have certain reproductive health needs unmet ^8^ ^,^ ^45^, including reports of coercive sterilization ^45^ ^,^ ^46^, frequently at childbirth ^8^ ^,^ ^46^ ^,^ ^47^ ^,^ ^48^, and a high rate of regret ^49^ ^,^ ^50^. A study comparing WLWHA and WNLWHA in the state of São Paulo, in 2013-2014, found no difference in the risk of sterilization between the two groups after adjusting for schooling, race/ethnicity and number of children, but observed a higher probability of sterilization of WLWHA at the time of delivery ^48^. A U.S. study with WLWHA found a low preference (3%) for sterilization in the post-HAART era, associated with a reduction in the mother-to-child transmission rate ^51^. Integrating sexual and reproductive health services and HIV services ^52^, removing institutional and structural barriers, and reducing stigma and discrimination are essential to free WLWHA’s reproductive choices from violence and coercion ^8^ ^,^ ^46^.

Some limitations must be highlighted. As pregnant women are not followed-up at INI/Fiocruz, information on perinatal outcome and mother-to-child transmission rate is unavailable. Besides, we had loss of information about the outcome in 6 (2.7%) participants. Absence of data on the intention to become pregnant, adequacy of method use, marital status at the time of pregnancy, and partner and family support limited assessing these factors. Finally, we were unable to assess the use of Dolutegravir (DTG) and its effect on pregnancy outcomes because DTG implementation became a 1st-line regimen in Brazil only in 2017 ^53^.

## Conclusion

We observed a significant reduction in pregnancy incidence in WLWHA after 2006, with a high rate of cesarean sections and a reduction in female sterilization. The association between pregnancy incidence and use of contraceptive methods and virological control markers suggests good integration between HIV/AIDS care services and sexual and reproductive health services. Conversely, the high rate of cesarean section delivery indicates the need to improve childbirth care.
